# HIV Prevention in Adolescents and Young People in the Eastern and Southern African Region: A Review of Key Challenges Impeding Actions for an Effective Response

**DOI:** 10.2174/1874613601812010068

**Published:** 2018-08-29

**Authors:** Kaymarlin Govender, Wilfred G.B. Masebo, Patrick Nyamaruze, Richard G. Cowden, Bettina T. Schunter, Anurita Bains

**Affiliations:** 1 Health Economics and HIV and AIDS Research Division, University of KwaZulu-Natal, Durban, South Africa; 2 School of Applied Human Sciences, University of KwaZulu-Natal, Durban, South Africa; 3 Department of Psychology, Middle Tennessee State University, Murfreesboro, United States of America; 4 *UNICEF, Eastern and Southern Africa Regional Office, Nairobi, Kenya*

**HIV Prevention in Adolescents and Young People in the Eastern and Southern African Region: A Review of Key Challenges Impeding Actions for an Effective Response**

The Open AIDS Journal, **2018**, 12: 53-67

## Correction

The corrections are provided and replaced online which is mentioned as under:

**Original:**

Current ethical and legal standards in most countries in the ESAR make it difficult to conduct health research with people under the age of 18 years [56] orphan 4. 4.

**Corrected:**

Current ethical and legal standards in most countries in the ESAR make it difficult to conduct health research with people under the age of 18 years [56] .

**Original:**

Some countries (*e.g.*, South Africa, Zimbabwe and Namibia) largely fund their HIV treatment programmes [77, 83] orphan 5, and domestic financing for HIV responses tend to be more secure and sustainable.

**Corrected:**

Some countries (*e.g.*, South Africa and Namibia) significantly fund their HIV treatment programmes. [77, 83].

